# Research on breast tumor segmentation based on the Mamba architecture

**DOI:** 10.3389/fonc.2025.1672274

**Published:** 2025-11-24

**Authors:** Weihao Wei, Jiacheng Wu, Guangming Shao

**Affiliations:** 1 Anhui University of Chinese Medicine, Hefei, China; 2 College of Medicine and Biological Information Engineering, Northeastern University, Shenyang, China

**Keywords:** breast tumors, medical image segmentation, Mamba, selective mechanism, hardware-aware algorithm

## Abstract

Medical image segmentation is fundamental for disease diagnosis, particularly in the context of breast cancer, a prevalent malignancy affecting women. The accuracy of lesion localization and preservation of image details are essential for ensuring the integrity of lesion segmentation. However, the low resolution of breast tumor B-mode ultrasound images poses challenges in precisely identifying lesion sites. To address this issue, this study introduces the Mamba architecture model, which combines three foundational models with the long-sequence processing model Mamba to develop a novel segmentation model for breast tumor ultrasound images. The selective mechanism and hardware-aware algorithm of the Mamba model enable longer sequence inputs and faster computing speeds. Moreover, integrating a complete chain of VMamba blocks into the basic model enhances segmentation accuracy and image detail processing capabilities. Experimental segmentation was performed on two benchmark ultrasound datasets (BUSI and BUS-BRA) using both the baseline and improved models. The results were compared using metrics such as Dice and IoU, with additional evaluations conducted under small-sample training conditions. This study is intended to provide guidance for the future development of medical image segmentation. Moreover, the experimental results demonstrate that the model incorporating the Mamba architecture achieves superior performance on breast ultrasound images.

## 1 Introduction

Tumors, which are caused by the aggregation of mutated cells into masses or growths, can be categorized into benign tumors that do not spread and malignant tumors that are uncontrollably cancerous (1). Breast cancer, one of the most commonly malignant tumors among women, is also one of the leading causes of cancer death in females. In the early stages, treatment is carried out through lumpectomy, with the goal of completely removing the tumor while preserving as much healthy tissue as possible. Therefore, the precision of tumor excision is a significant challenge in this surgery, and for patients with unclear margins, there is a high probability of requiring a second excision, which may cause patients to miss the best treatment time and increase their psychological burden. For the high incidence of positive cancer margins after breast tumor excision, accurate tumor localization is key to overcoming this challenge. Ultrasound detection is considered the best method for examining breast tumors due to its non-radiation and non-invasive medical imaging approach (2). However, the frequency of the ultrasound equipment and probe directly affects image quality and lesion display, thereby influencing the diagnostician’s judgment (3), leading to missed diagnoses and misdiagnoses, which highlights the importance of early precise detection for successful treatment.

Conventional diagnostic methods relying on subjective judgments have limitations and risks of misdiagnosis (4). Medical image segmentation is a crucial technology in medical image processing (5, 6), essential for disease diagnosis, treatment planning, and evaluating treatment outcomes. Accurate segmentation delineates diseased and normal tissue boundaries, providing precise anatomical and pathological information for clinical decision-making (5). However, due to the inherent limitations of ultrasound imaging, such as poor contrast and the variability in the appearance of tumors, the development of reliable and effective segmentation algorithms still faces significant challenges. Deep convolutional neural networks (DCNNs) (7) have revolutionized this field by automatically extracting key visual features relevant to disease diagnosis from extensive medical image datasets (8, 9). Recent advancements in medical image segmentation, notably the UNet deep-learning network, have shown remarkable potential in segmenting and classifying breast tumor images (10). UNet’s exceptional performance and adaptable network structure have made it a focal point in research (11).

To further enhance segmentation models, researchers are exploring novel network architectures like dense connections (12), residual blocks (13), and attention mechanisms (14). Kumari et al. (15) utilized a neural network with a dense connection known as Densely Connected Convolutional Network (DCCN) to identify deep liver irregularities; (16) introduced a deep learning architecture (MRFB-Net) that leverages an attention-based pooling decoder module to enhance the segmentation of uterine fibroids in preoperative ultrasound images. However, common CNN models face limitations in their ability to model long-range interactions, and Transformers are constrained by their quadratic computational complexity, making them less than satisfactory for processing breast ultrasound images. This has led to the emergence of State Space Models (SSM) (17, 18), represented by Mamba, as a promising solution. The Mamba model excels not only in modeling long-range interactions but also in maintaining linear computational complexity. It specifically improves the S4 state space model through selective mechanisms and hardware-aware algorithms, excelling in processing long-sequence data with its unique features. By integrating the cross-scan module (CSM) into the visual state space model (VMamba), Mamba enhances its applicability to computer vision tasks by spatially traversing the domain (17). (19) proposed the Shuffle-Reshuffle Gradient Mamba (SRGM) tailored for MMIF, and designed the Local and Global Gradient Mamba (LGGM) to extract modality-specific features while retaining rich spatial details. (20) introduced Semi-Mamba-UNet, which integrates a pure vision-based Mamba-based U-shaped encoder-decoder architecture with the traditional CNN-based UNet into a semi-supervised learning (SSL) framework and tested it on the ACDC and PROMISE12 medical imaging datasets. (21) introduce Edge-Mix enhanced Mamba (EM-Mamba) for kidney segmentation, which is designed to capture global and local information from multi-scales. EM-Mamba leverages SegMamba as its backbone, utilizing Mamba’s efficiency in extracting long-range dependencies. Although Transformer models excel at global modeling, their self-attention mechanism requires a computational complexity that is quadratic with respect to the image size (22), which becomes particularly evident in the task of medical image segmentation that demands dense predictions. Building on these advancements, our goal is to enhance long-sequence data processing by integrating Mamba into foundational models like UNet++ (23) and DeepLabv3+ (24), aiming to improve breast ultrasound image segmentation.

Integrating the VMamba block (VSS) (25) from the Mamba model into other networks enhances the model’s medical image segmentation performance. The VSS features a unique selective mechanism and hardware-aware algorithm, offering significant advantages in processing long-sequence data. By adaptively selecting crucial information for processing, the Mamba model avoids redundant computations, thereby enhancing computational efficiency. Additionally, its hardware-aware algorithm enables seamless adaptation to diverse hardware platforms, further expediting the model’s inference process. Our research focuses on demonstrating the notable benefits of incorporating the Mamba structure into an image segmentation model for breast tumor image segmentation and classification tasks. This integration enables precise differentiation between tumor tissues and normal breast tissues, resulting in high-precision image segmentation. Specifically, we integrated the VMamba module into the encoder of the model, thereby effectively capturing the multi-scale spatial features and global contextual cues of breast ultrasound images. We conducted extensive experiments on the BUSI and BUS-BRA datasets using various metrics, and the results demonstrated that the models incorporating the VSS block achieved higher segmentation accuracy for breast ultrasound images compared to the original models. This enhancement enables precise segmentation of diverse breast tumors and their complex boundary structures. Such accuracy provides valuable support for clinicians, advancing the clinical application and scientific exploration of artificial intelligence technology in medical image processing, particularly in addressing challenges related to breast tumor image processing.

## 2 Mamba model structure

Mamba, a state space model (SSM), shares the capability of transformers in extracting global features from lengthy sequences. However, Mamba distinguishes itself through its selective mechanism and hardware-aware algorithm, resulting in an inference speed five times faster than that of Transformers. Notably, Mamba’s computational complexity and memory usage scale linearly with input sequence length, allowing it to process sequences of millions in length. In contrast, Transformers exhibit a time and space complexity of *O*(*n*^2^), highlighting Mamba’s ability to markedly alleviate GPU memory and computing resource demands during the training of long-sequence text models (17). Mamba integrates the SSM architecture with the multi-layer perceptron (MLP) block within the Transformer framework. SSM serves to characterize state representations and forecast their subsequent states given specific inputs. The structured state space sequence model operates on the following principle:

(1) $$h^{\prime }\left(t\right)=Ah\left(t\right)+Bx\left(t\right)$$

(2) y(t)=Ch(t)+Dx(t)

In this context, *h*(*t*) denotes the current state variable, *A* signifies the state transition matrix, *x*(*t*) represents the input control variable, and *B* indicates the impact of the control variable on the state variable (26). Furthermore, *y*(*t*) denotes the system output, while *C* signifies the influence of the current state variable on the output. The state and output equations imply that the state at time step *t* is predicted from the preceding state. By incorporating past information in the sequence and the input from the prior state, the system’s future states can be anticipated. The *A* state transition matrix plays a crucial role in updating the sequence state by incorporating skip connections. These connections directly combine the previous input with the output sequence, thereby improving feature extraction. To tackle the challenge of context sequence dependencies, SSM utilizes Hierarchical Positional Pointers (HiPPO) for long-range dependencies. By employing function approximation, SSM achieves the optimal solution (27) of the matrix *A*, enabling the retention of a more extensive historical record.

Selection Mechanism: The conventional SSM model excels in processing structured input data. In contrast, Mamba introduces a selective mechanism that parameterize the SSM input. This mechanism selectively compresses historical data, filters out extraneous 18 information, and preserves essential long-term memory. Consequently, Mamba addresses the challenge faced by traditional models in managing fluctuations or disorder in input sequences, thereby ensuring that parameters influencing sequence interactions adapt to the input dynamics. Specifically, a new learnable parameter step size Δ represents the stage resolution, sampling the continuous input signal over time to obtain discrete output, which is realized by solving the ordinary differential Equation 2 and performing a direct discretization operation. Then, by sampling with step size Δ (i.e., $d\tau {❘}_{t_{i}}^{t_{i+1}}={\text{Δ}}_{i}$), $h\left(t_{b}\right)$ can be discretized by Equation 4.

(3) $$h\left(t_{b}\right)=e^{A\left(t_{b}-t_{a}\right)}h\left(t_{a}\right)+e^{A\left(t_{b}-t_{a}\right)}\int_{t_{a}}^{t_{b}}B\left(\tau \right)u\left(\tau \right)e^{-A\left(\tau -t_{a}\right)}\;d\tau$$

(4) $$h_{b}=e^{A\left({\text{Δ}}_{a}+\ldots +{\text{Δ}}_{b-1}\right)}\;(h_{a}+\sum_{i=a}^{b-1}B_{i}u_{i}e^{-A\left({\text{Δ}}_{a}+\ldots +{\text{Δ}}_{i}\right){\text{Δ}}_{i}})$$

In addition, performing zero-order hold processing on parameters A, B to obtain $\bar{A}=\text{exp }(\text{Δ}A)$, $\bar{B}={\left(\text{Δ}A\right)}^{-1}\left(\text{exp }(\text{Δ}A\right)-I)\text{Δ}B$, ultimately converting the continuous SSM to a discrete SSM, thus updating Equations 1 and 2 to 3 and 4.

(5) $$h_{k}=\bar{A}h_{k-1}+\bar{B}x_{k}$$

(6) $$y_{k}=Ch_{k}$$

In contrast to the fixed spacing between input and output elements in conventional copy tasks, selective copying involves adjusting token positions based on content-specific reasoning to eliminate extraneous information. As illustrated in Equation 7, this process incorporates an additional linear layer in each matrix computation to selectively filter input control and state variables, thereby enhancing reasoning efficiency and augmenting data throughput. Enhancement of the *B* matrix affecting input, the *C* matrix influencing state, and the Δ time-size parameter enables the model to discern the content of individual tokens, which represent the smallest meaningful units understood and generated by the model. The dimensions of *B, C* and Δ can be extended by incorporating functions *s_B_*(*x*), *s_C_*(*x*) and *s*_Δ_(*x*). The introduction of the selection mechanism addresses the limitation of SSM in screening signals across time.

(7) $$\begin{array}{c} s_{B}\left(x\right)=Linear_{N}\left(x\right),s_{C}\left(x\right)=Linear_{N}\left(x\right),\\ s_{\text{Δ}}\left(x\right)=Linear_{D}\left(x\right),\tau_{\text{Δ}}=softplus \end{array}$$

### Hardware-optimized algorithm

The Mamba algorithm utilizes a multi-threaded parallel scanning approach that leverages the associative law for executing out-of-order computations and aggregating outcomes. In this method, each sequence involves updating the state $H_{i}$ according to Equation 8, where it is computed by multiplying the previous state with a matrix $\bar{A}$ and adding the current input $X_{i}$ multipied by $\bar{B}$. The parallel scanning process integrates segmental sequence computation and iteration to achieve its objectives.

(8) $$H_{i}=\bar{A}H_{i-1}+\bar{B}X_{i}$$

Notably, the cyclic convolution mode enables bypassing the initial fixed state (*B, L, D, N*), leading to the utilization of a more efficient 3*a* convolution kernel (*B, L, D*) and significantly enhancing computational performance.

The state $H_{i}$ is exclusively operational within the memory hierarchy. To mitigate memory bandwidth constraints, the kernel fusion technique is employed to diminish GPU memory occupancy, thereby substantially enhancing training velocity. The utilization of Flash Attention technology alters the computation outcomes sequentially inscribed in DROM to batch writing from DRAM, thereby curtailing the frequency of redundant read and write operations (28). Consequently, is substitutedfor the initial ( $\bar{A},\bar{B}$) with a scale of (B,L,D,N) and fed into the high-speed Static Random-Access Memory (SRAM). To mitigate the need for storing intermediate states during backpropagation, the utilization of recomputation technology is imperative. This approach aims to minimize memory usage by recalculating intermediate states during the backward pass, rather than storing them when loading input from High Bandwidth Memory (HBM) to SRAM. By implementing this technique, the selective scanning layer can achieve a level of memory efficiency akin to that of the high-speed attention Transformer.

## 3 Data processing

The study leveraged data from two publicly available datasets: BUSI (29) and BUS-BRA (30). The BUSI dataset comprises breast ultrasound images and their corresponding label images, collected from 600 women aged 25 to 75 in 2018. Each original image is paired with a tumor image (mask), with benign and malignant samples typically featuring one or two lesions. As a result, the labels outlining the lesion areas may require overlapping to consolidate multiple lesions into a single label. The BUS-BRA dataset includes 1875 anonymized breast ultrasound images from 1064 patients, with 722 benign and 342 malignant tumors. It provides BI-RADS assessments, manual segmentations, and 5- and 10-fold cross-validation partitions for standardized evaluation of CAD systems. The detailed information of the dataset is shown in **Table 1**.

**Table 1:** Introduction of dataset.

Case	BUSI	BUS-BRA
Number of Images	780	1875
Benign	437	1268
Malignant	210	607
Normal	133	–
Annotation Information	Masks	Masks and BI-RADS classification
Dataset Characteristics	Smaller	Larger, suited for extensive training

Adequate data is essential for effectively training deep learning networks to prevent underfitting and subpar classification performance (31). To bolster model robustness, a substantial volume of high-quality datasets is necessary (32). However, obtaining medical image data is intricate, necessitating the expansion of existing public datasets through data augmentation techniques. In our approach, we employ online augmentation, randomly rotating and mirror-flipping each image and its corresponding label in the dataset to enhance the model’s generalization capabilities. Furthermore, we enhance image quality by applying linear transformations to address the indistinct edges characteristic of ultrasound images in the dataset in function (9), thus suppressing the Hausdorff dimension inflation caused by ultrasonic speckle noise. Because the scanning position varies across breasts, the collected ultrasound images exhibit inconsistent sharpness and brightness. We therefore perform dynamic contrast normalization as defined in Equation 9: the gray-level histogram of each image is first computed, its intensity bins are used to derive an adaptive weight, and the image contrast is adjusted accordingly, yielding a standardized dataset.

(9) O(i,j)=α*I(i,j)+b     0≤i<H,0≤j<W

Here, *H* and *W* denote the height and width of the input image, *I*(*i,j*) represents a pixel point in the input image, *O*(*i,j*) for the output image; by adjusting the size of parameters *a,b* to achieve transformation of the image grayscale range, thereby adjusting the image contrast.

## 4 Research on ultrasound breast tumor image segmentation based on mamba architecture

This study integrates the Mamba model with different segmentation network architectures to enhance the performance of medical image segmentation. By incorporating the VSS block featuring the Mamba model into diverse segmentation networks, improvements in segmentation accuracy are achieved. Evaluation on a dataset and comparison of segmentation outcomes of the fused models demonstrate that the integration of the Mamba structure accelerates computation while preserving long-term data information.

### 4.1 Analysis of the VMamba block

**Figure 1:**
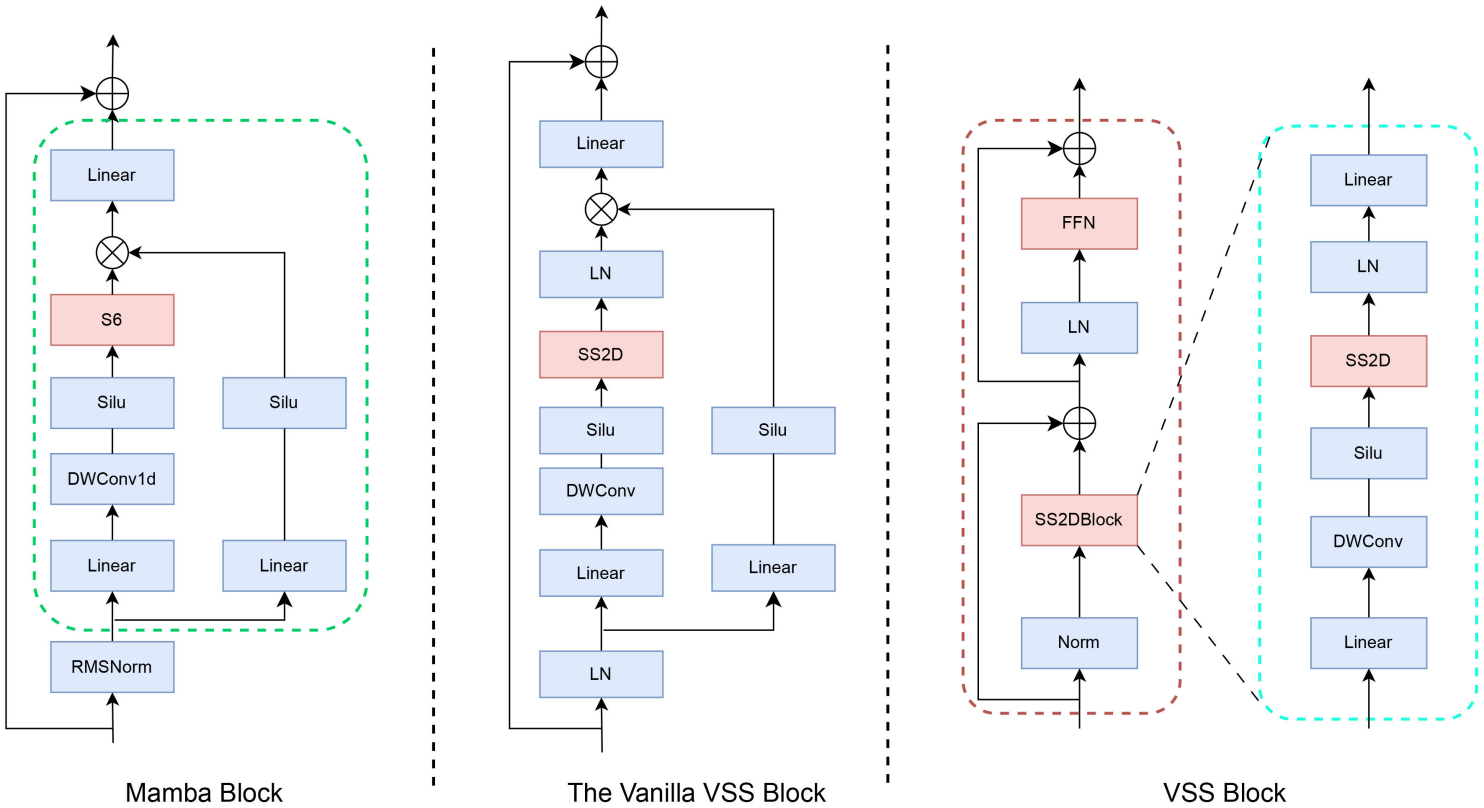
Structures of the Mamba block (left), the Vanilla VSS block (middle), and the VMamba (VSS) block.

**Figure 1** illustrates the architecture of the VMamba block, comprising an H3 block and a gated MLP. The H3 block embodies a selective SSM (independent sequence transformation) state-space model. Simplifying the H3 structure involves amalgamating linear attention and MLP blocks, stacking them uniformly, and enabling controlled expansion of the model dimension. The Mamba architecture is constructed by iteratively replicating this block, incorporating residual connections and standard normalization interchangeably. To mitigate gradient vanishing, a residual term is introduced in conjunction with the gated MLP. The VMamba block is limited to extracting features from semantic data like text and cannot handle image data. To address this limitation, Yue et al. (33) substituted the S6 module in the VMamba block with the SS2D module, which is designed to process image data using the VSS block. This modification resulted in the creation of the VSS block.

Following layer normalization, the VSS block comprises two branches. One branch employs a 3×3 depthwise convolutional layer for feature extraction. Initially, the input undergoes processing in a linear layer, a depthwise separable convolution, and an activation function before entering the two-dimensional selective scanning (SS2D) module for further feature extraction. Subsequently, feature normalization is applied, followed by element-wise multiplication with the output from the alternate branch to merge the pathways. A linear layer is then utilized to blend the features, which are combined with a residual connection to yield the VSS module output. The second branch includes a linear mapping layer followed by a SiLU activation layer to compute the multiplicative gating signal. Notably, the key distinction from the standard VSS block lies in replacing the S6 module with the SS2D module, enabling adaptive selective scanning for 2D visual data. This design choice opts for a more compact structure without the fully connected phase, resulting in denser stack blocks within the same depth constraints.

### 4.2 Construction of the VM-UNet++ model

**Figure 2:**
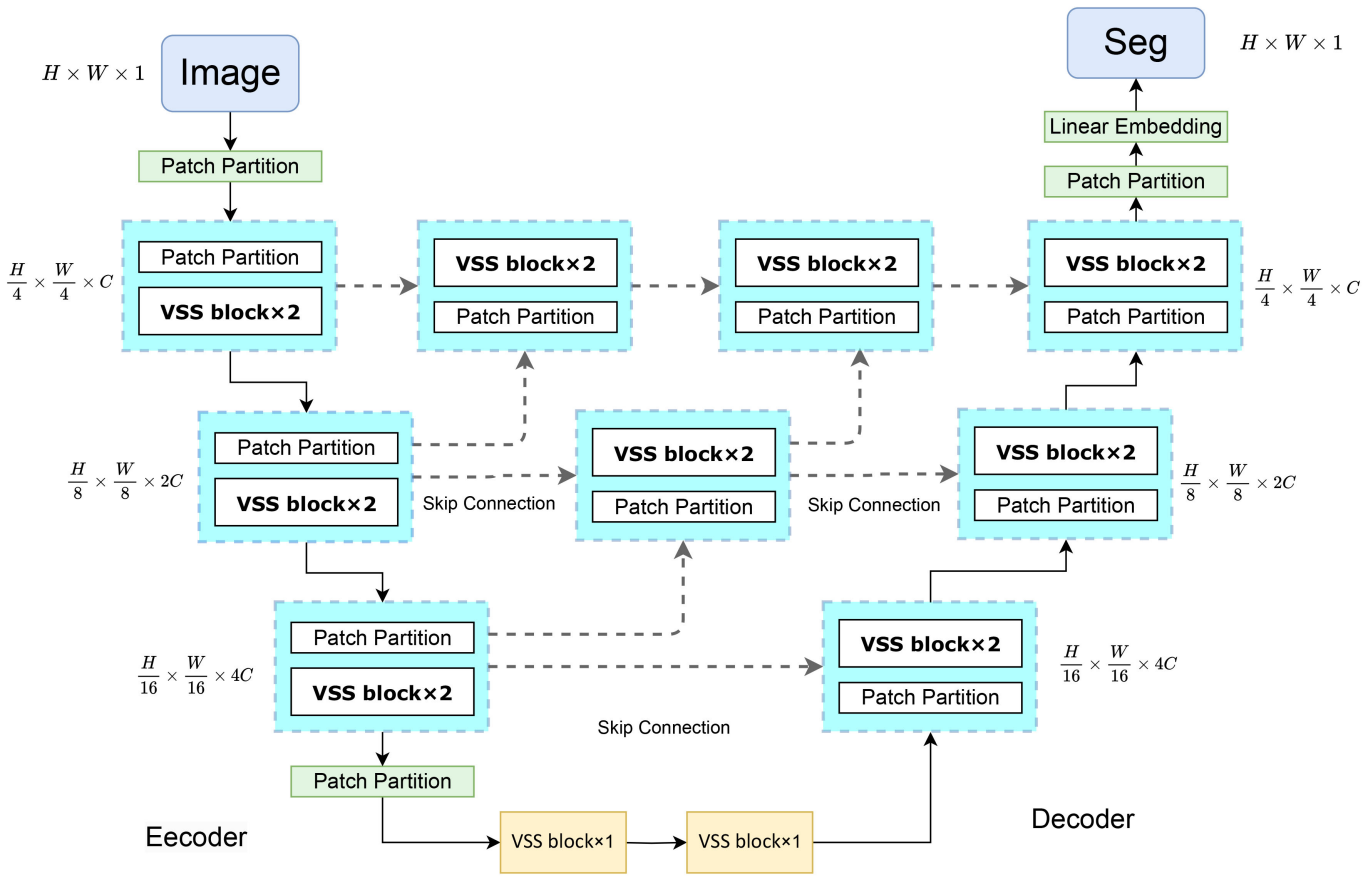
Structure diagram of VM-UNet++.

**Figure 2** illustrates the architecture of VM-UNet++. This design integrates the U-Net framework with the VSS block to construct the encoder and decoder components. The U-Net features a symmetrical U-shaped configuration comprising an encoder for feature extraction, a decoder for feature fusion, and skip connections to mitigate gradient vanishing (15). Within the decoder’s upsampling phase, skip connections are employed post each convolution to link with the downsampled encoder features at the corresponding level and lower-level features, thereby diminishing gradient vanishing and preserving more spatial detail features. The VM-UNet++ configuration encompasses a patch embedding layer, an encoder, a decoder, a final projection layer, and skip connections. Initially, the input image is transformed into a one-dimensional sequence of H/4×W/4×C via the patch and linear embedding layers. The encoder incorporates multiple VSS blocks and patch merging layers to extract token features, diminish height and width, and augment dimensionality (34). The decoder mirrors the encoder’s structure, with the patch merging layer substituted by a patch expansion layer to enhance height and width while reducing dimensionality, thereby generating outputs with consistent feature sizes. Ultimately, the linear projection layer restores the channel count to align with the input resolution. A densely connected network is introduced during the upsampling phase, where the convolution output of the preceding layer is added to each subsequent layer, forming local Unet networks within each segment. This approach fuses low-resolution features from upsampling with high-resolution features from downsampling to retain both spatial detail features and global information. The densely connected structure of the VM-UNet++ model facilitates straightforward network depth augmentation to bolster learning capacity during construction. Moreover, it permits a moderate depth reduction through network pruning strategies without compromising the original network architecture.

### 4.3 Construction of Deep-VMamba

**Figure 3:**
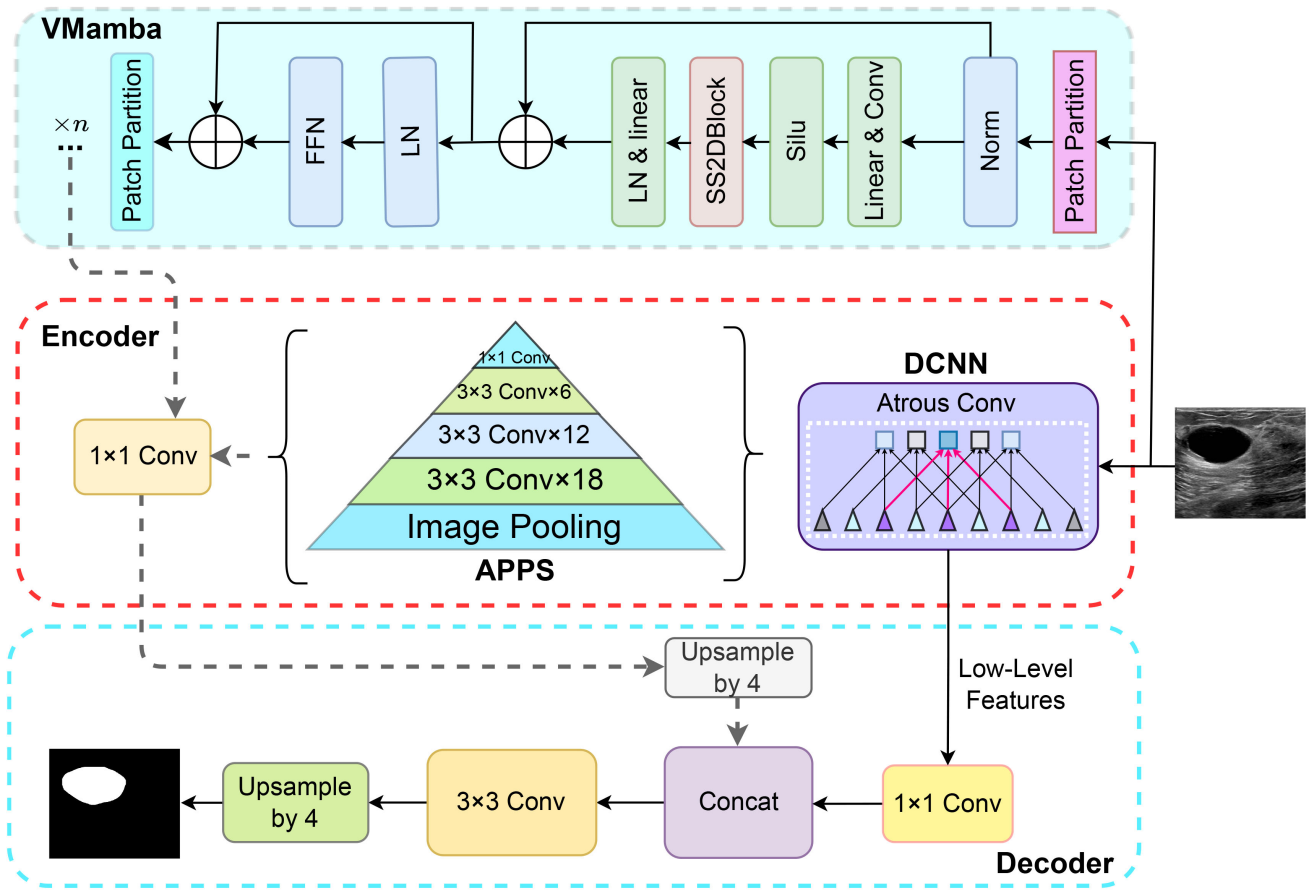
Structure of the Deep-VMamba model.

DeepLabV3+ comprises an encoder and a decoder, incorporating Atrous convolution, depthwise separable convolution, Atrous Spatial Pyramid Pooling (ASPP), and fully convolutional networks (31). As illustrated in **Figure 3**, this study integrates the Mamba structure into the DeepLabV3+ architecture. The VMamba block, fused with the encoder output of DeepLabV3+, enhances the delineation of tumor lesions in ultrasound breast images by capturing finer details and edge information. The incorporation of the VMamba block supplements global information to the original DeepLabV3+ segmentation, thereby expanding the network’s receptive field without compromising feature retention, facilitating more comprehensive malignant tumor segmentation. Additionally, the encoder segment of DeepLabV3+ encompasses a complete feature extraction and sampling branch, preserving all feature extraction capabilities while augmenting the model’s proficiency in feature extraction from images and processing extended sequences, without compromising its original functionality.

### 4.4 Construction of the SAM-VMamba model

**Figure 4:**
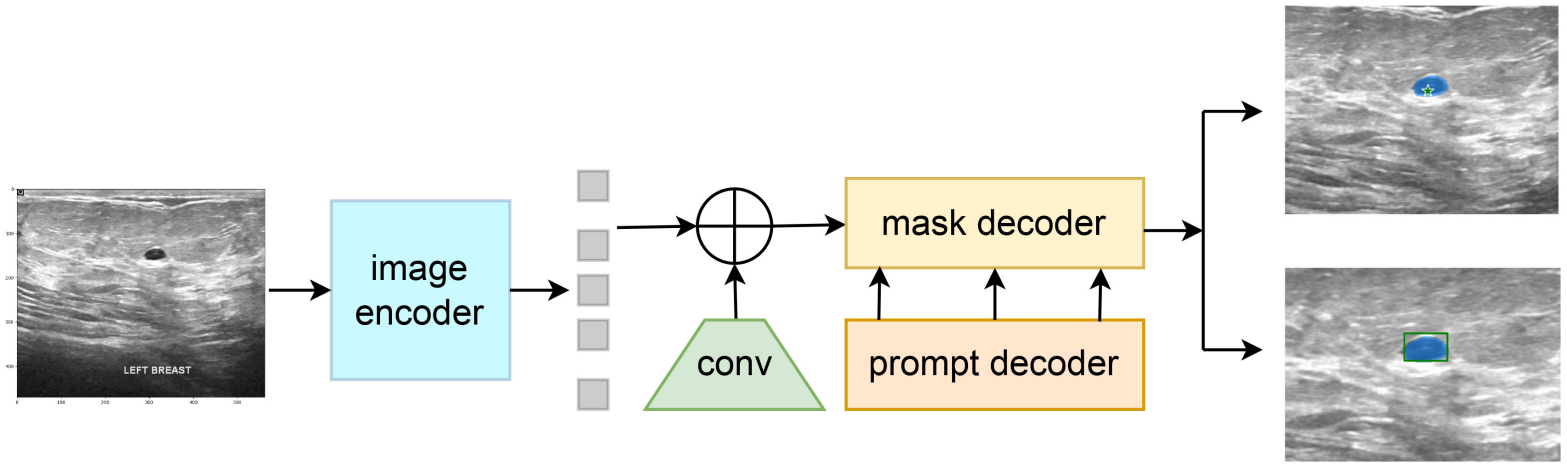
Structure of the SAM model.

The Segment Anything Model (SAM) model is tailored for a novel image segmentation assignment, trained on a dataset of 11 million images with over one billion masks. Moreover, SAM can segment images based on various prompts such as points, boxes, and text, without the need for retraining on specific datasets. Its efficient design and training facilitate zero shot transfer to new image distributions and tasks, which has garnered widespread attention. For instance, Ma and Wang et al. (35) proposed MedSAM for general medical image segmentation. This model, trained on a meticulously constructed dataset, is capable of achieving desirable performance. However, the limited scale of the assembled dataset and the modality imbalance issue restrict MedSAM’s performance on ultrasound images. The MSA method proposed by Wu et al. (36) significantly enhances image segmentation performance by freezing the pre-trained parameters of SAM and inserting adapter modules at specific locations. As shown in **Figure 4**, the SAM model comprises an image encoder, a prompt encoder, and a mask decoder, the SAM model employs a prompting approach to segment user-specified points. Users can provide prompt information through user-defined points, bounding boxes, and randomly circled regions. Furthermore, free-form text prompts are utilized to present initial results. Notably, the prompt encoder of the SAM model can effectively segment desired objects based on user prompts, thereby enabling targeted area segmentation. For the segmentation of breast ultrasound images, Tu et al. (37) proposed an innovative SAM adapter (BUSSAM), which migrates the SAM framework to the field of breast ultrasound image segmentation through adaptation techniques, and validated its feasibility and effectiveness.

**Figure 5:**
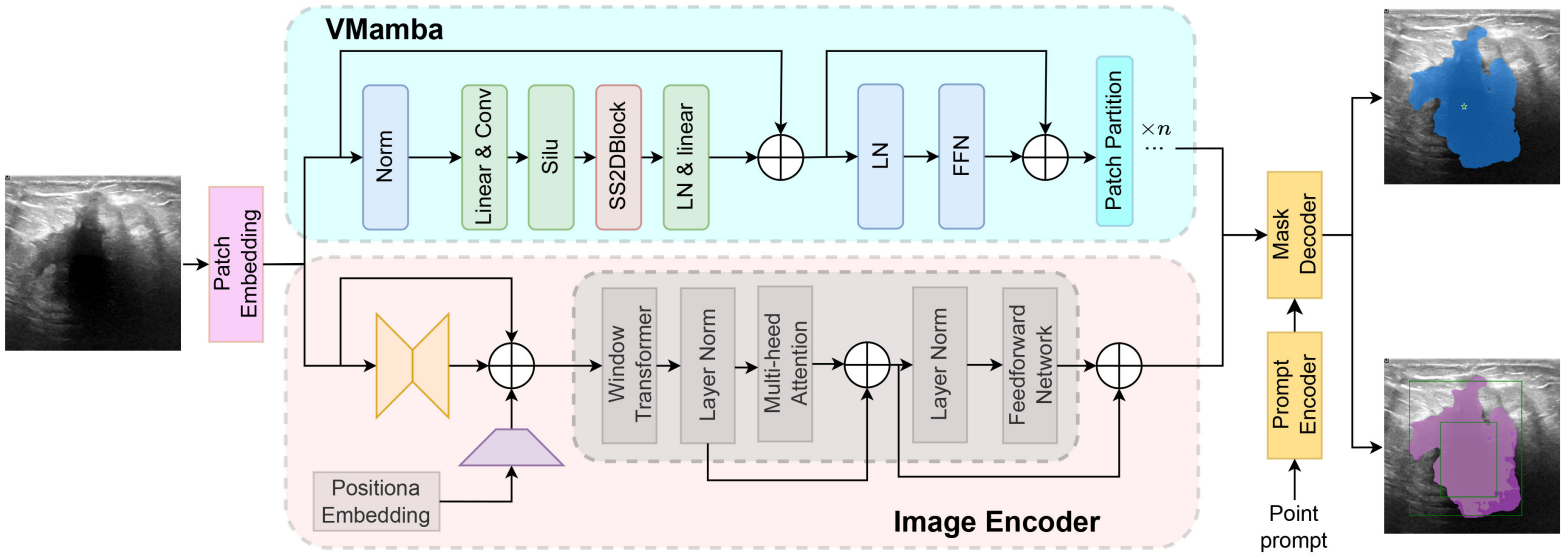
Structure of the SAM-VMamba model.

Although the SAM demonstrates evident effectiveness for the segmentation of the vast majority of natural images, it faces challenges when dealing with fine medical images due to the inherent low resolution and complexity of medical imaging, leading to suboptimal performance in zero-shot segmentation scenarios. Therefore, few-shot training becomes crucial for achieving superior performance in practical applications. Moreover, considering the limitations of SAM in global attention, our study incorporates the VMamba block into the SAM model framework to enhance its capabilities. As shown in **Figure 5**, the VMamba block, situated alongside the ViT within the SAM image encoder, facilitates the processing of extended input sequence data for a more comprehensive contextual understanding. Illustrated in **Figure 5**, the model comprises an image encoder, a prompt encoder, and a mask decoder. Following the Patch Embedding step in the image encoder, the VMamba block operates in parallel to convert the upper layer’s output tokens into a linear vector with long-range memory. This vector is then fused with the Transformer block output and subjected to two convolutions (Neck layer) to generate the Image Embedding, which subsequently serves as input for the mask decoder.

The enhanced comprehension of extended sequences by the Mamba model enables SAM-VMamba to establish improved global connections and achieve enhanced segmentation performance. Additionally, the integration of a selective mechanism and hardware-aware algorithm in SAM-VMamba expedites model training and implementation without incurring additional time costs, thereby substantially decreasing the time and resources needed for training deep segmentation models. Moreover, by integrating the generalization and pre-training capabilities of the SAM model, the SAM-VMamba model is able to achieve accurate segmentation effects with only a small number of breast ultrasound image training samples.

## 5 Experimental results and analysis

### 5.1 Experimental environment

The model proposed in our study is deployed and trained on the RTX A6000 GPU, with all experiments conducted using the same hardware device. The experiments utilize the PyTorch 2.2.0 deep learning framework and Python 3.11.5 programming language, with GPU computation supported by the CUDA 12.2 architecture. The batch size is set to 24, with a maximum of 100 training epochs, and the AdamW optimizer is employed in conjunction with the CosineAnnealingLR learning rate scheduling strategy for model optimization. Additionally, the proposed SAM-VMamba network is initialized using the pre-trained weights of SAM’s ViT-B.

### 5.2 Evaluation indicators

The Dice Coefficient (DICE) metric assesses model performance in segmentation tasks by quantifying the overlap between predicted and ground-truth regions. In medical image analysis, the DICE coefficient is commonly employed to evaluate neural network models in tasks like lesion detection and tissue segmentation. The formula for calculating the DICE coefficient is defined by **Equation 10**.

(10) $$DICE=\frac{2\left|TP\right|}{2\left|TP\right|+\left|FP\right|+\left|FN\right|}$$

The MIoU metric assesses the correspondence between predicted outcomes and true labels in semantic segmentation tasks. It is computed as the mean of the Intersection over Union (IoU) for individual categories. IoU represents the ratio of the intersection to the union of predicted and actual values, reflecting the degree of overlap. A higher IoU signifies improved segmentation accuracy, indicating a greater overlap between areas. The calculation is given by **Equation 11**.

(11) $$IoU=\frac{\left|TP\right|}{\left|TP\right|+\left|FP\right|+\left|FN\right|}$$

Precision is defined as the proportion of pixels that are correctly identified as the true lesion area. It represents the ratio of true positive pixels to the sum of true positive and false positive pixels, expressed as: $\frac{\left|TP\right|}{\left|TP\right|+\left|FP\right|}$. Accuracy represents the proportion of correctly identified image pixels, that is, the ratio of breast tumor and non-breast tumor areas to the total number of pixels (mask), expressed as: $\frac{\left|TP\right|+\left|TN\right|}{T+P}$. Recall, also known as the sensitivity, is the proportion of the actual lesion area that is identified in the image. It represents the size of the true positive cases relative to the entire lesion area, expressed as: $\frac{\left|TP\right|}{\left|TP\right|+\left|FN\right|}$.

### 5.3 Experimental results

**Table 2** summarizes the training hyper-parameters and computational performance of each model. Where, throughput denotes the maximum number of training samples the model can process per second, while total multiply-adds signify the computational burden of the model during a single forward propagation. The exceptional long-sequence processing capabilities of Mamba are confirmed through an assessment of the computational efficiency of output images. This evaluation involves comparing parameters, processes, and throughput during both training and inference to gauge generalization performance. Results indicate that models incorporating Mamba exhibit consistent performance across various input image sizes. For instance, at an input resolution of 512 × 512, Unet++ achieves the highest throughput among baseline models, while DeepLabV3+ demonstrates the highest throughput per epoch during training. Despite higher computational load compared to baseline models at the same input size, the integration of the Mamba structure allows for increased throughput capacity, enabling the retention and processing of longer data sequences, thereby enhancing comprehensive image data processing. Moreover, while the integrated models maintain relatively high inference speeds (higher throughput than baseline models) at a resolution of 512×512, their computational load escalates significantly, surpassing that of baseline models and indicating limited generalization capability. In terms of computational efficiency, current SSM-based vision models typically exhibit superior throughput only with large-scale inputs and high resolutions.

**Table 2:** Parameter table on the 512^2^ image.

Model	#param	FLOPs	Throughput	Train throughput	Total mult-adds
Unet	24.4M	31.3G	75.38	57.69	31.26
Unet++	26.1M	73.7G	71.12	33.72	73.53
DeepLabV3+	22.4M	31.7G	73.71	58.44	31.54
VM-UNet	27.4M	16.4G	48.87	25.46	310.02
SAM-Med2D	221.9M	303.5G	21.28	24.10	259.31
VM-UNet++	27.4M	27.2G	47.03	30.27	443.97
Deep-VMamba	27.8M	121.2G	64.15	46.90	43.28
SAM-VMamba	236.5M	259.8G	27.73	35.35	303.26

Based on **Tables 3** and **4**, it is evident that traditional models do not achieve highly accurate segmentation of ultrasonic breast tumor images. The Unet model, for instance, exhibits relatively low performance with Dice coefficients of 81.92% and 82.10%, and IoU values of 69.53% and 73.52% across the two datasets. In contrast, models incorporating the VMamba block demonstrate a significant improvement in segmentation metrics compared to their original counterparts. Notably, the SAM-VMamba model, which integrates the VMamba block, achieves the highest performance with Dice scores of 90.62% and 90.25%, and IoU values of 82.55% and 82.54%. This improvement can be attributed to the inherent challenges posed by breast ultrasound images, characterized by low clarity and predominantly dark tones, leading to a diminished signal-to-noise ratio. Given that breast tumors occupy a small portion of the image, there is a risk of lesion oversight and misjudgment. Furthermore, the indistinct boundary between breast tumors and normal tissues, coupled with blurred lesion edges lacking distinctive features against the background, contributes to reduced segmentation accuracy. Moreover, the uniform grayscale distribution in the images results in minimal variations in pixel intensities, thereby compromising texture and detail resolution. However, the integration of Mamba facilitates the capture of prolonged sequential information, enabling more comprehensive breast tumor segmentation and enhanced edge delineation.

**Table 3:** Presents a comparative evaluation of the segmentation outcomes of the models on BUSI.

Model	DICE(%)	IoU(%)	Precision(%)	Accuray(%)	Recall(%)
Unet	81.92	69.53	86.33	97.78	78.53
Unet++	82.76	70.81	86.98	97.81	79.32
DeepLabV3+	83.61	71.97	88.05	97.92	79.74
VM-UNet	83.56	74.13	84.58	97.60	84.43
SAM-Med2D	89.13	81.16	89.45	98.56	87.43
VM-UNet++	84.89	75.59	86.57	97.99	87.61
Deep-VMamba	84.24	71.24	88.26	97.84	79.21
SAM-VMamba	90.62	82.55	88.44	98.08	92.05

**Table 4:** Presents a comparative evaluation of the segmentation outcomes of the models on BUS-BRA.

Model	DICE (%)	IoU(%)	Precision(%)	Accuray(%)	Recall(%)
Unet	82.10	73.52	87.30	97.51	84.27
Unet++	84.88	76.28	87.02	97.43	85.71
DeepLabV3+	86.43	77.98	87.50	97.74	88.24
VM-UNet	85.69	77.19	87.95	97.59	86.48
SAM-Med2D	88.68	80.05	97.49	98.35	81.76
VM-UNet++	86.35	78.01	88.95	97.63	86.61
Deep-VMamba	87.16	79.01	87.82	97.82	88.99
SAM-VMamba	90.25	82.54	96.19	98.58	85.43

**Figure 6:**
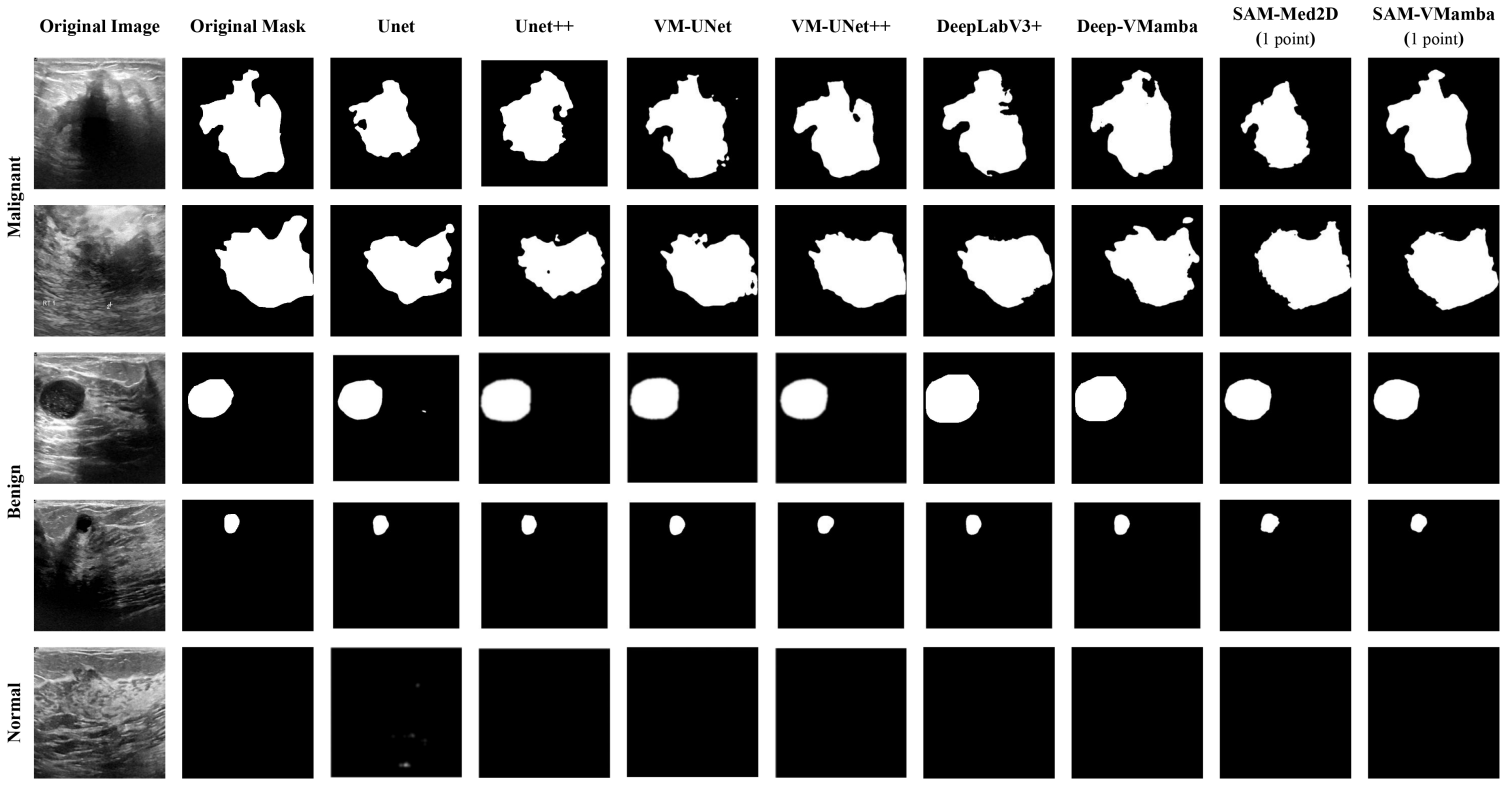
Comparison of image segmentation effects on the BUSI dataset.

**Figure 7:**
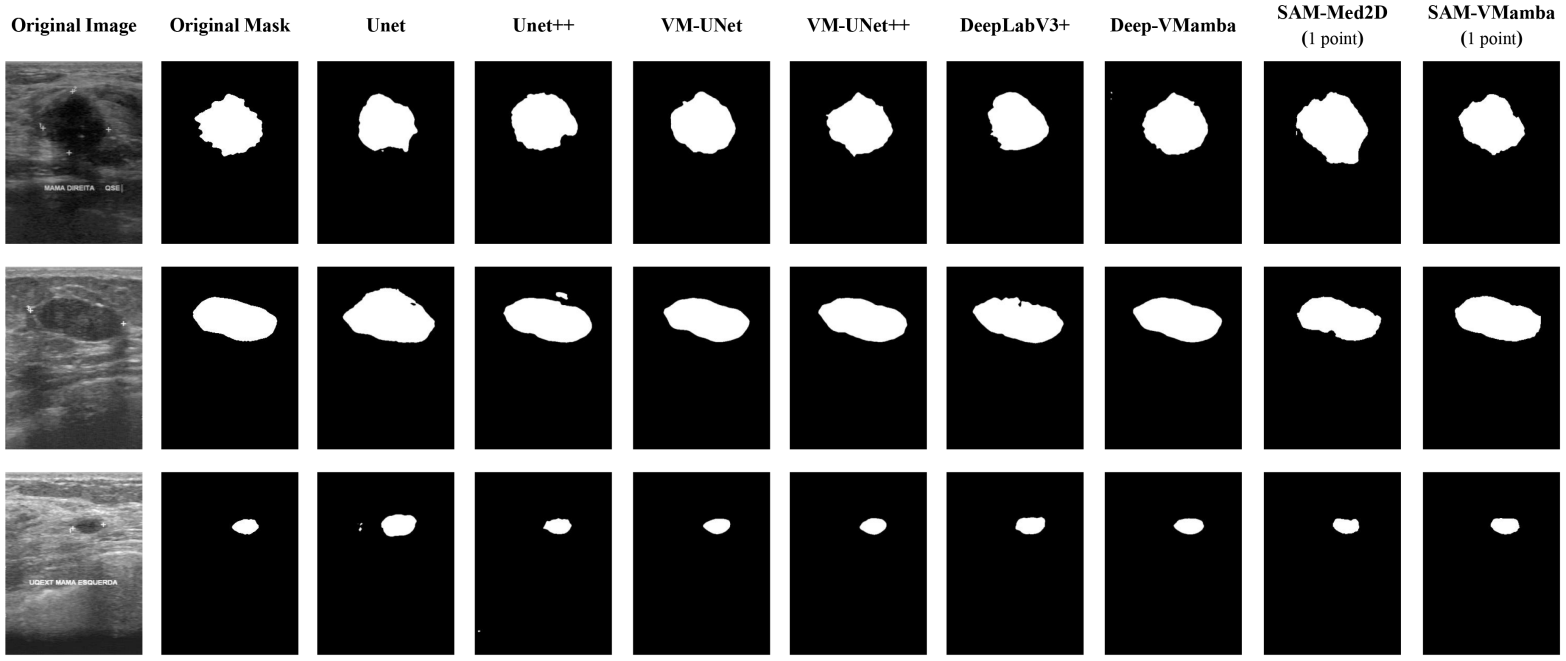
Comparison of image segmentation effects on the BUS-BRA dataset.

**Figure 8:**
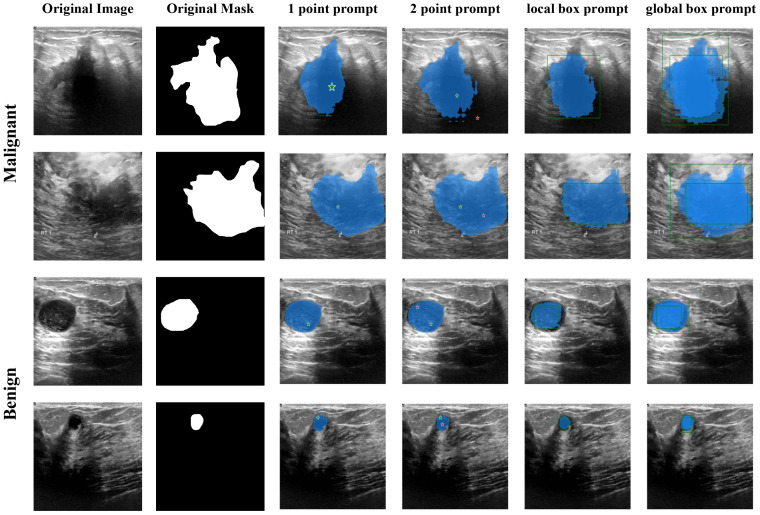
Segmentation results of SAM-VMamba.

**Figures 6** and **7** visualize the actual segmentation results of each model on breast tumors. Traditional models exhibit poor performance in segmentation due to the limitations of their structure. The pooling layers and downsampling operations used in network training result in the loss of partial information as the network depth increases and size decreases. In contrast, during upsampling, only a basic addition operation is conducted on high-resolution images from the downsampling layer, leading to the loss of crucial “deep-layer” feature information. However, Mamba, characterized by its capacity for ultra-long sequence processing and memory within the integrated model, preserves more spatial details, thereby yielding superior segmentation outcomes. SAM-VMamba demonstrates superior performance with small-sample data due to its integration of the SAM segmentation model. Furthermore, as illustrated in **Figure 8**, the distinctive prompt encoder of SAM enables precise regional segmentation of images, enhancing its practical utility by eliminating the need to process redundant image components.

**Figure 9:**
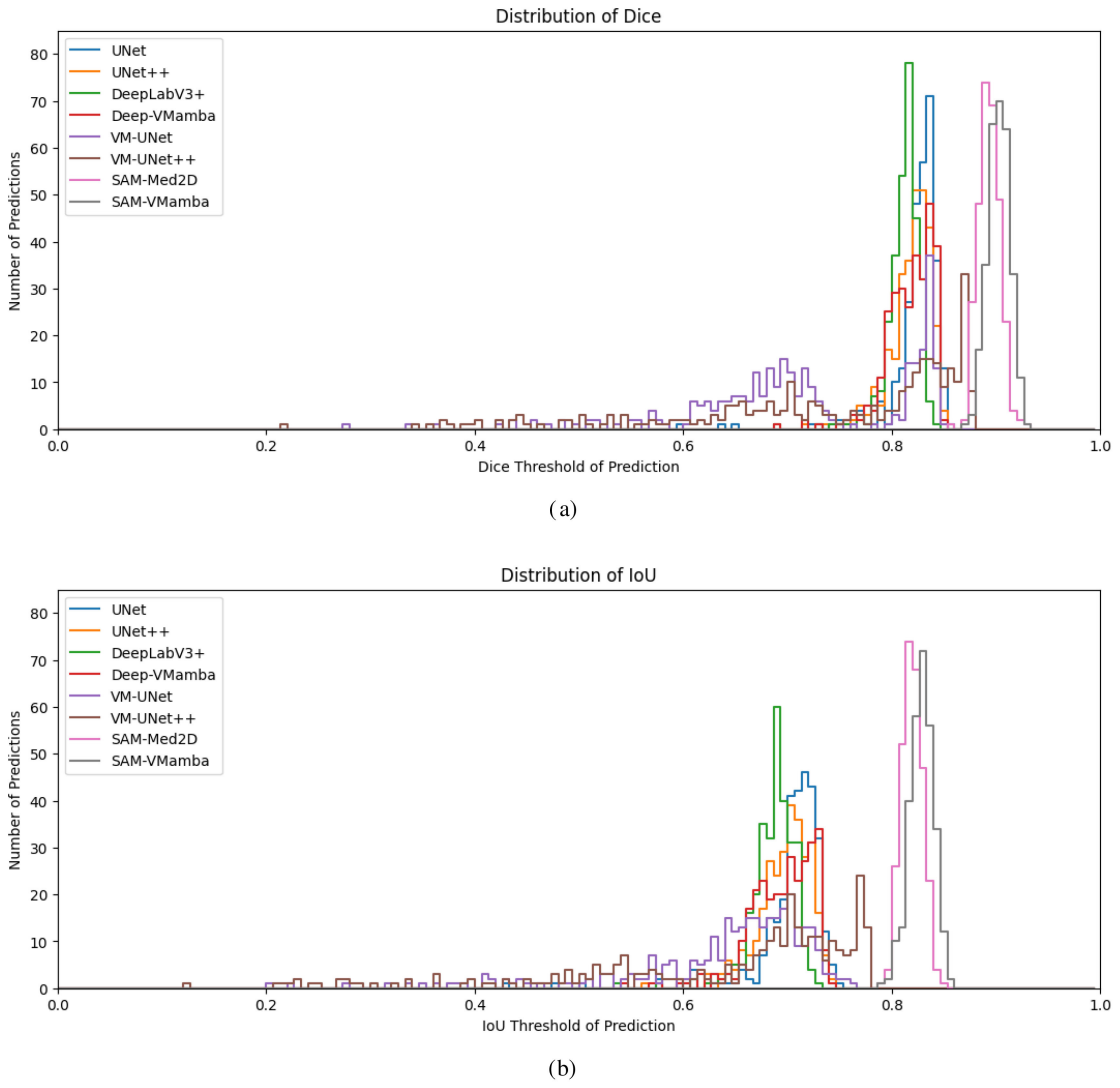
Cumulative distributions of dice and IoU under 300 predictions. **(a)** Dice, **(b)** IoU.

**Figures 9a, b** depict the cumulative distribution of prediction effects for each model based on 300 segmentation predictions of breast ultrasound images. The segmentation outcomes of conventional models such as U-Net predominantly cluster around 0.8 for Dice and 0.7 for IoU. The integration of the Mamba model notably enhances the overall segmentation performance, yielding higher accuracy metrics compared to the baseline models. Particularly noteworthy is the superior segmentation efficacy of the SAM model surpassing that of its counterparts. The SAM model demonstrates heightened segmentation accuracy and data concentration, indicative of its robust stability. The primary reason lies in the prompt encoder mechanism of the SAM model and its pretraining on large-scale datasets, which enable superior adaptation and handling of out-of-domain datasets. In contrast, baseline models suffer from a substantial loss distance between their initialized weights and the optimal solution, requiring large sample sizes and extensive iterations to reduce this gap, thereby leading to unstable extrapolation in prediction distributions. Moreover, models augmented with the Mamba structure further enhance the multi-scale spatial decomposition of breast tumor images and the modeling of intra-scale feature dependencies, thereby facilitating the extraction of tumors with varying shapes and types.

**Figure 10:**
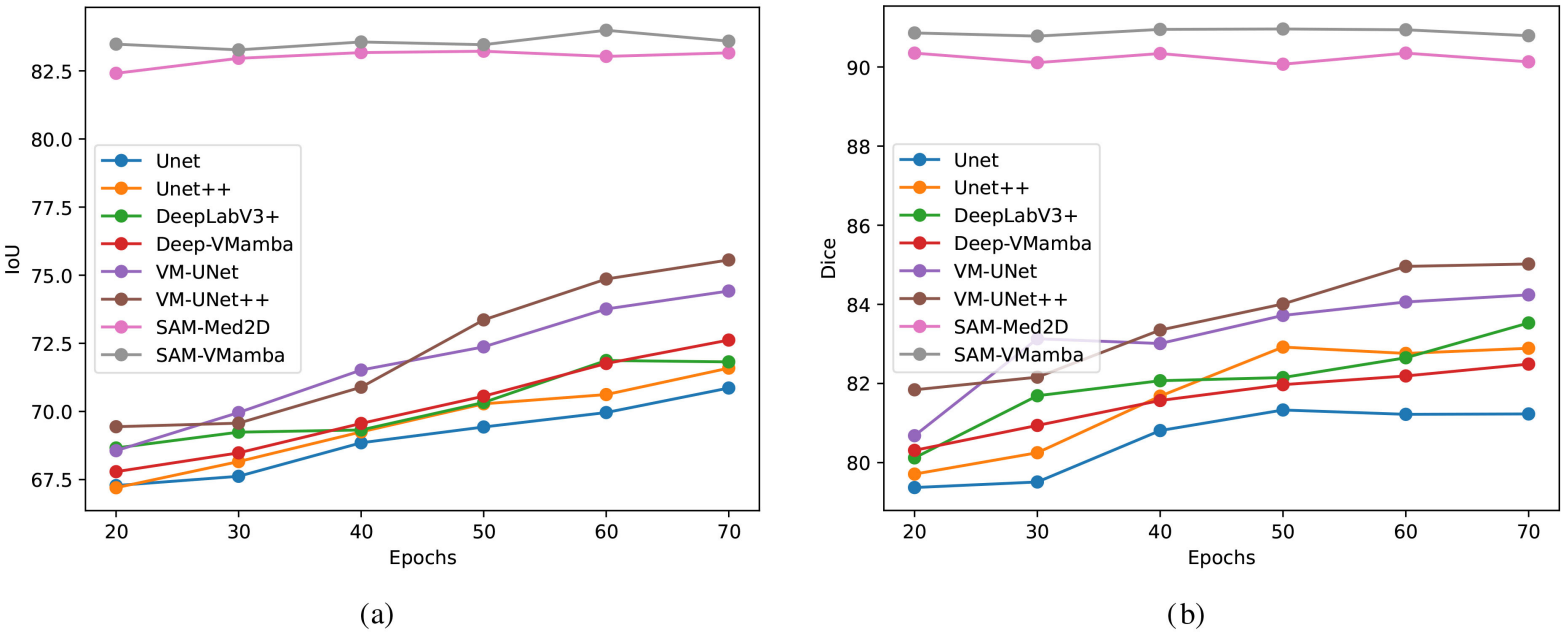
Comparison of segmentation accuracies among different models trained with a low number of iterations. **(a)** IoU, **(b)** Dice.

The **Figures 10a, b** illustrates notable enhancement in the model’s performance with the integration of the Mamba structure compared to the original model. Specifically, the incorporation of this structure significantly improves the segmentation performance of the model on breast ultrasound images with long sequences. With an increase in the number of training iterations, conventional models like Unet exhibit some degree of enhancement. However, the integrated Mamba model surpasses the performance of individual traditional models in overall improvement. Particularly noteworthy is the superior segmentation performance of the SAM model compared to other models, attributed to its pre-training on a large dataset of millions of images. This model requires only a limited number of training epochs to achieve a stable and optimal performance level.

**Figure 11:**
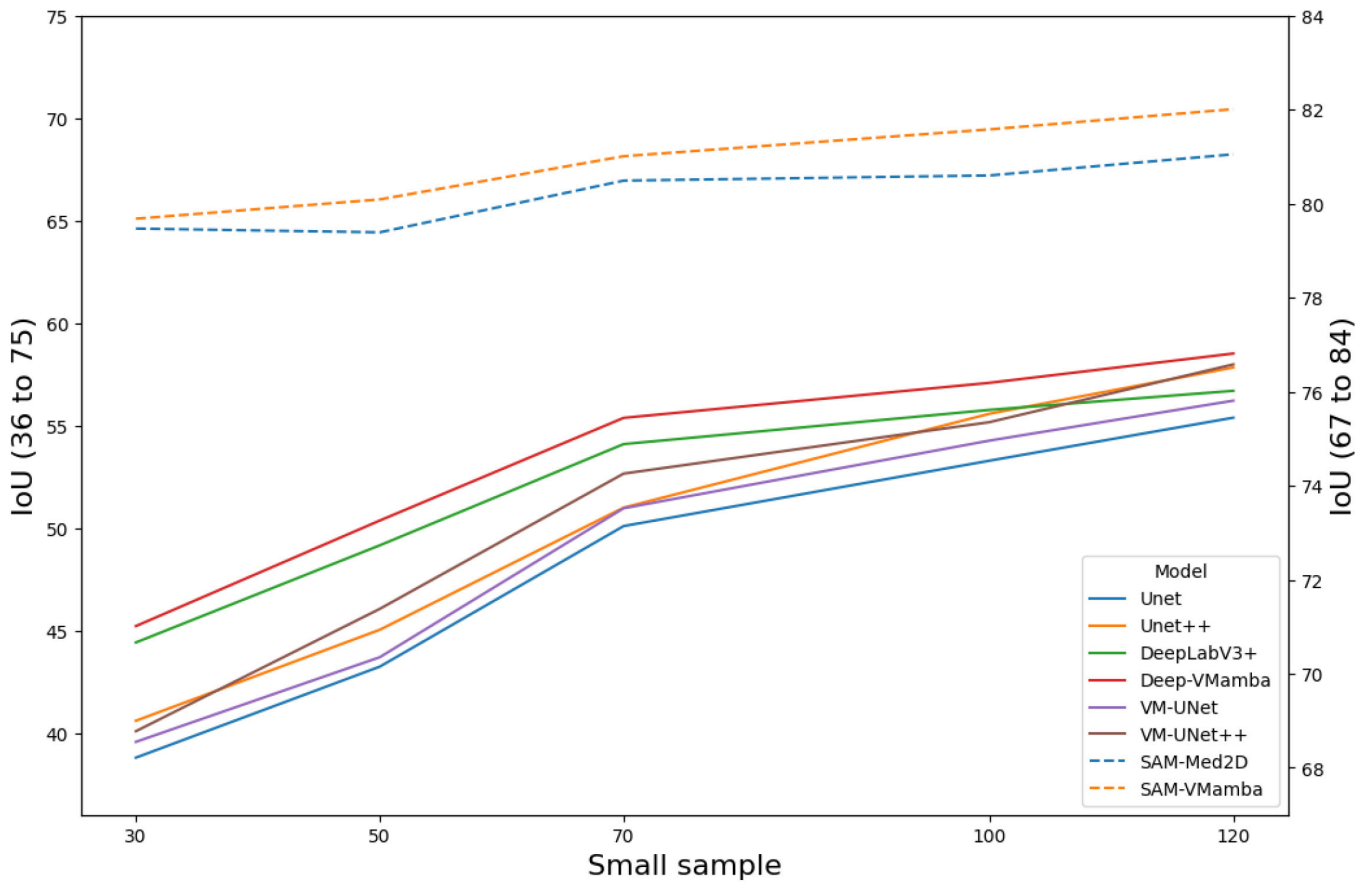
Comparison of segmentation accuracy (IoU) of each model on small samples.

**Figure 11** presents the performance of all models under few-sample testing conditions. Findings indicate that when trained on a small dataset, the model incorporating VMamba blocks generally outperforms the baseline model in segmentation accuracy. The primary reason is that breast tumors exhibit a relatively low signal-to-noise ratio and indistinct features, which forces baseline models to rely on large sample averaging to suppress noise. In contrast, Mamba compresses two-dimensional spatial sequences into fixed-dimensional state vectors, inherently embedding a Gaussian–Markov smoothing mechanism that provides natural denoising. These advantages collectively enable the Mamba-enhanced model to achieve an average improvement of 9.12% in the IoU metric. Remarkably, despite being pretrained on extensive data, the SAM model exhibits sustained high segmentation performance in the context of limited-sample training, maintaining an IoU value of approximately 80%.

## 6 Conclusions

In this investigation, we enhanced the image segmentation performance of the original model for breast tumor ultrasound images by integrating the Mamba structure. Comparative analysis with conventional models like Unet, DeepLabV3+, and Unet++ revealed the superior performance of the model incorporating the VMamba block across various evaluation metrics, including the DICE coefficient, MIoU, Precision, and Recall. Benefiting from the global attention capability of Mamba, the enhanced model is able to simultaneously capture multi-scale global dependencies and better focus on the details of breast tumor segmentation. Experimental results show that incorporating Mamba into the model yields average improvements of 3.07% and 5.11% in Dice and IoU on the BUSI dataset, and 2.89% and 3.26% on the BUS-BRA dataset. Notably, the SAM-VMamba achieved the highest segmentation accuracy and quality, with Dice scores of 90.25% and 90.62% on the BUSI and BUS-BRA datasets. These outcomes signify the model’s success in accurately localizing and distinguishing boundaries of breast tumors.

## Data Availability

The original contributions presented in the study are included in the article/supplementary material. Further inquiries can be directed to the corresponding author.
